# Influence of Prosthetic Material Properties and Implant Number on Stress Distribution in Implant–Bone Systems Under Bruxism Loading: A Finite Element Study

**DOI:** 10.3390/biomimetics11020089

**Published:** 2026-01-27

**Authors:** Derya Aslan, İsmail Hakkı Korkmaz, Nuran Yanıkoğlu, Abdullah Tahir Şensoy

**Affiliations:** 1 Department of Prosthodontics, Faculty of Dentistry, Atatürk University, 25240 Erzurum, Turkey; derya.aslan@atauni.edu.tr (D.A.); ndinckal@atauni.edu.tr (N.Y.); 2 Department of Mechanical Engineering, Faculty of Engineering and Architecture, Erzurum Technical University, 25050 Erzurum, Turkey; ismail.korkmaz@erzurum.edu.tr; 3 Faculty of Mechanical Engineering, Delft University of Technology, Mekelweg 2, 2628 CD Delft, The Netherlands; 4 Department of Oral and Maxillofacial Surgery, Erasmus University Medical Center, Doctor Molewaterplein 40, 3015 GE Rotterdam, The Netherlands; 5 Department of Biomedical Engineering, Faculty of Engineering and Natural Sciences, Samsun University, 55420 Samsun, Turkey

**Keywords:** bruxism, dental implants, implant-supported prostheses, prosthetic materials, finite element analysis

## Abstract

This finite element study compared the effects of prosthetic superstructure material and supporting implant number on stresses in implants, multiunit abutments, and restorations, and on peri-implant bone strains under bruxism-like loading. Two posterior mandibular models representing missing left FDI 34–36 were generated: a 2-implant configuration (implants at 34 and 36) and a 3-implant configuration (implants at 34, 35, and 36), each restored with a three-unit implant-supported fixed bridge. For each configuration, three superstructure materials were simulated: cobalt–chromium (Co–Cr), polyetheretherketone (PEEK), and monolithic zirconia (MZ). Static parafunctional loads were applied as a 500 N oblique load (30° to the implant long axis; 125 N to each buccal cusp) and a 1000 N vertical load applied to the central fossae. Cortical bone generally exhibited higher strain than trabecular bone, and the maximum cortical principal strain under vertical loading averaged approximately 5800 μɛ. The highest implant von Mises stress occurred in the first molar implant of the 2-implant MZ model under oblique loading, while the maximum under vertical loading was 236 MPa (also 2-implant MZ). Prosthetic peak stresses reached 184 MPa under vertical loading (3-implant PEEK composite–veneered model) and 233 MPa under oblique loading (2-implant MZ), with a minimum of 51 MPa in the 3-implant PEEK framework under vertical loading. Overall, increasing implant number reduced the stress/strain values, and MZ showed comparatively higher stress and strain levels.

## 1. Introduction

Improving the success of implant therapy requires a careful evaluation of the biomechanical principles and appropriate prosthetic planning. From a biomechanical perspective, implant design, surface characteristics, diameter, length, number, angulation, occlusal load transmission, and the selection of suitable prosthetic superstructure materials play a crucial role in the long-term performance of implant-supported restorations [1].

Occlusal overload, particularly when combined with lateral forces, is considered one of the main etiological factors associated with implant failure, marginal bone loss, and prosthetic complications [2]. Bruxism, defined as clenching and/or grinding of the teeth during daytime or nighttime, is a common parafunctional activity and a major contributor to occlusal overload [3]. Bruxism-related occlusal forces have been associated with temporomandibular joint disorders, tooth wear, hard tissue loss, periodontal support loss, restoration fractures, and various complications in implant therapy [4,5]. Due to the magnitude, direction, and repetitive nature of these forces, bruxism poses a significant biomechanical challenge for implant-supported prosthetic rehabilitations.

The selection of restorative materials is a key factor influencing the long-term stability and clinical success of implants exposed to parafunctional forces. Prosthetic superstructure materials affect the manner in which functional and parafunctional stresses are transmitted to the prosthetic components, implant, and bone–implant interface [6,7,8]. Previous studies have demonstrated that rigid, non-polymeric materials with a high elastic modulus lack shock-absorbing capacity, leading to increased stress accumulation at the bone–implant interface [9]. In contrast, polymeric materials such as polyetheretherketone (PEEK), due to their force-absorbing properties, may promote a more uniform distribution of occlusal loads and reduce stress concentration in peri-implant bone. These characteristics are particularly relevant in patients with bruxism, where excessive parafunctional loading is expected [10]. Titanium-based materials, particularly commercially pure titanium and Ti–6Al–4V, remain the most widely used implant materials due to their favorable biocompatibility and mechanical performance. In restorative materials research, tribological, mechanical, and thermal properties are increasingly evaluated together, and a recent ranking study of nano-hydroxyapatite-filled dental composites (R-method) highlights how multi-criteria property trade-offs determine overall material performance, supporting the rationale for comparing alternative superstructure materials in biomechanical analyses [11].

Despite the clinical relevance of bruxism, there are limited in vitro studies investigating the biomechanical behavior of implant-supported prostheses fabricated from materials with different elastic moduli under parafunctional loading conditions. Therefore, finite element analysis (FEA) has been widely used as an effective numerical method to evaluate stress and strain distribution in implants, abutments, prosthetic components, and peri-implant bone tissues under complex loading scenarios, including bruxism-related forces.

Bruxism-related occlusal loading is highly variable across individuals and even within the same patient over time, depending on episode frequency, duration, jaw position, and the relative contribution of tonic versus phasic masticatory muscle activity. Accordingly, “force magnitude” estimates in the literature span a wide range and are difficult to generalize because measurement approaches differ substantially (e.g., polysomnography vs. portable EMG, device thresholds, and outcome definitions). A recent systematic review and meta-analysis on portable instrumental assessment of sleep bruxism emphasized the high heterogeneity between studies and the presence of outliers even when using bruxism event thresholds, underscoring that a single representative loading level cannot be assumed for all bruxers [12]. In parallel, a recent systematic review and meta-analysis summarizing bruxism treatment outcomes reported inconsistent bite-force-related findings across protocols and follow-up periods, further supporting the notion that bruxism loading characteristics are not uniform across cohorts [13]. Therefore, finite element studies commonly adopt high-end static loads and unfavorable directions to approximate worst-case parafunctional scenarios rather than relying on a unique typical bruxism force.

Accordingly, the aim of this study was to investigate, using finite element analysis, the stress and strain distribution generated by bruxism-related parafunctional forces in implant-supported fixed prosthetic restorations fabricated with superstructure materials of different elastic moduli, namely cobalt–chromium (Co–Cr), polyetheretherketone (PEEK), and monolithic zirconia (MZ). The first hypothesis was that the use of prosthetic materials with lower elastic moduli would reduce stress and strain values in the implant, surrounding bone, and prosthetic restoration under bruxism loading. The second hypothesis was that increasing the number of implants would further reduce stress and strain in these structures under bruxism-related parafunctional loading conditions.

## 2. Materials and Methods

In this study, the effects of parafunctional forces on different implant numbers and prosthetic superstructure materials (cobalt–chromium (Co–Cr), polyetheretherketone (PEEK), and monolithic zirconia (MZ)) were investigated. Finite element analysis (FEA) was employed to evaluate potential damage in bone structures by determining principal strain values.

**Figure 1:**
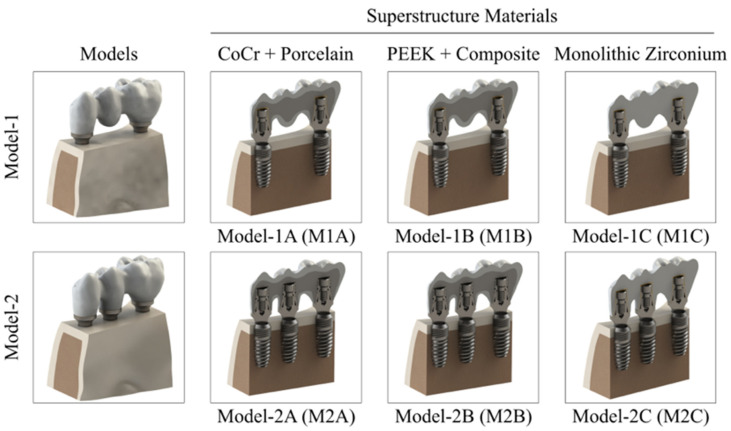
Finite element models of two-implant-supported (Model 1) and three-implant-supported (Model 2) fixed prosthetic restorations with different superstructure materials.

Within this framework, six different finite element models were constructed. Three-dimensional models of the mandibular bone, implants, multiunit (MU) abutments, titanium-base (Ti-base) abutments, and prosthetic superstructure materials were generated for the study (Figure 1). Three-dimensional solid component modeling and finite element analysis were performed using SolidWorks software (v2019; Dassault Systèmes, Waltham, MA, USA) and ANSYS Workbench (ANSYS Inc., Canonsburg, PA, USA), respectively. All analyses were conducted on a workstation equipped with an AMD Ryzen 9 5950X 3.70 GHz processor (Advanced Micro Devices Inc., Santa Clara, CA, USA), 1 TB NVMe M.2 hard drive (Samsung Electronics Co., Suwon, Gyeonggi-do, Republic of Korea), 64 GB RAM (G.SKILL International Enterprise Co., Taipei City, Taiwan), and running the Windows 10 Ultimate operating system.

Implant-supported fixed prosthetic restorations were designed for a posterior mandibular region with missing left mandibular first premolar, second premolar, and first molar teeth (FDI: 34, 35, and 36). The two-implant-supported design was defined as Model 1, whereas the three-implant-supported design was defined as Model 2. According to the prosthetic superstructure materials, the models were classified as cobalt–chromium porcelain (Group A), PEEK–composite (Group B), and monolithic zirconia (Group C) (Figure 1). In all models, bone-level implants with a diameter of 4.1 mm and a length of 10 mm (Nucleoss, İzmir, Türkiye) were used.

All implant models, titanium-base (Ti-base) abutments, multiunit abutments, and intermediate components used in the study were assembled using SolidWorks(v24, Dassault Systems, Waltham, USA) software. The cement-layer thicknesses were defined to represent clinically realistic cement spaces by considering that an ideal cement gap for marginal adaptation in CAD/CAM crowns is typically in the range of approximately 25–50 μm and that the occlusal internal gap tends to be higher than the axial/lateral regions (approximately 90–144 μm), therefore, 0.05 mm was assigned to the lateral regions and 0.1 mm to the occlusal region [14]. Initially, the dental implant–abutment–screw–cement components were assembled, after which the corresponding crowns and bone structures were positioned accordingly. The assembled models were exported in Parasolid format and imported into the Static Structural analysis module of ANSYS Workbench. The mechanical properties of the materials used in the analysis are presented in Table 1. All materials were defined as linear elastic, homogeneous, and isotropic. Although modeling anisotropic and heterogeneous bone structures is more accurate biomimetically, taking these into account requires substantially more detailed material characterization and increases model complexity and uncertainty. Therefore, in this study, which performed a comparative analysis under the same conditions, the commonly adopted simplification of defining bone structures as elastic, homogeneous, and isotropic materials was used [15,16].

As reported in similar studies [23], frictional contact was defined between metallic components to simulate realistic clinical conditions, whereas bonded contact was applied at the implant–bone and crown–cement interfaces. A friction coefficient of 0.3 was assigned to the frictional contacts [24], and the Augmented Lagrange formulation was used for contact resolution.

For mesh generation, SOLID187 and SOLID186 tetrahedral elements with 10 nodes were employed to generate the finite element models. These element types are suitable for irregular geometries and exhibit quadratic displacement behavior. The average number of elements used in the numerical models was approximately 500,000. In this study, mesh convergence was evaluated to ensure the reliability of the finite element mesh and to achieve mesh-independent results. During the convergence procedure, mesh metrics were monitored regularly, and the mesh was refined to minimize element distortion by maintaining the skewness values below 0.30 and improve the element integrity by keeping the mesh element quality above 0.70. With these thresholds satisfied, the absence of meaningful changes in stress/strain outputs in the critical regions (implant, multiunit abutment, and peri-implant bone) across successive mesh refinements supported that the model had reached mesh independency.

**Figure 2:**
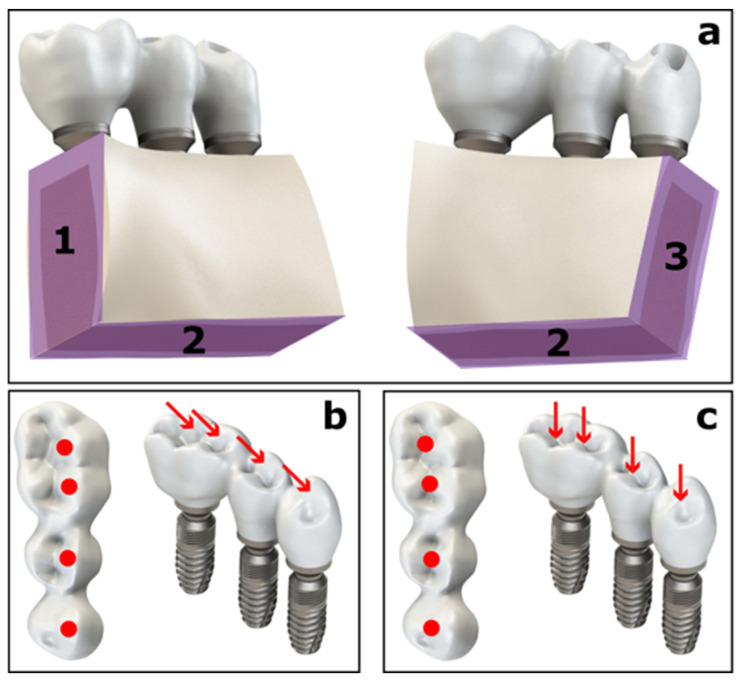
Boundary conditions and parafunctional loading configuration used in the finite element models: (**a**) constraint regions of the mandibular bone, and combined (**b**) oblique and (**c**) vertical parafunctional load.

After mesh generation, boundary conditions were defined in accordance with previously published studies. As illustrated in Figure 2, the selected bone surfaces (surfaces 1, 2, and 3) were constrained in all directions using fixed support boundary conditions.

Based on data reported in the literature [20,25,26,27,28], masticatory forces generated during parafunctional activity were applied. Oblique static parafunctional loads with a total magnitude of 500 N were applied at a 30° inclination to the buccal cusps of the left mandibular first premolar, second premolar, and first molar, with 125 N applied to each cusp (Figure 2b). In addition, a total vertical static parafunctional load of 1000 N was applied to the central fossae of the same teeth in a separate loading condition (Figure 2c) [15]. Oblique and vertical loads were analyzed as independent loading scenarios to simulate bruxism-related parafunctional activity [16,29]. Since the aim of the study was to compare different prosthetic materials under the same loading conditions, dynamic loading conditions and fatigue conditions were not taken into account.

Six finite element models were constructed based on two implant configurations and three prosthetic superstructure materials. Each model was analyzed under two different loading boundary conditions (vertical and oblique), resulting in a total of twelve finite element analyses. Because frictional contact was defined between metallic components, the analyses were solved using a nonlinear approach. Solutions were obtained under static boundary conditions using the Static Structural module. For all models, the solution converged with an average of 16 iterations.

## 3. Results

**Figure 3:**
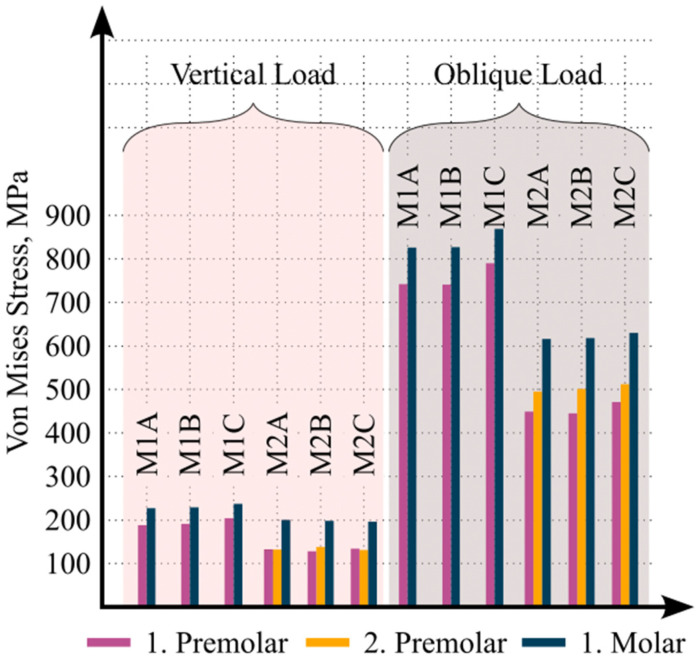
Equivalent von Mises stress results obtained from the FEA under different loading and material conditions across all components.

The maximum von Mises stress values generated in the implants under vertical loading are shown in Figure 3. When the maximum von Mises stress values among the models were compared, the highest value was observed in the first molar implant of Model M1C, reaching 236 MPa. In all models, stress was concentrated predominantly in the cervical region of the implant and gradually decreased toward the apical region. The highest stress concentrations were located on the lingual surface of the implant neck.

Similarly, the maximum von Mises stress values in the implants under oblique loading are presented in Figure 3. Among all models, the highest von Mises stress value was recorded in the first molar implant of Model M1C, measuring 869 MPa. In all models, stress concentration was primarily observed in the cervical region of the implant, with decreasing values toward the apex. The highest stress concentrations under oblique loading were localized on the distobuccal surface of the implant neck.

**Figure 4:**
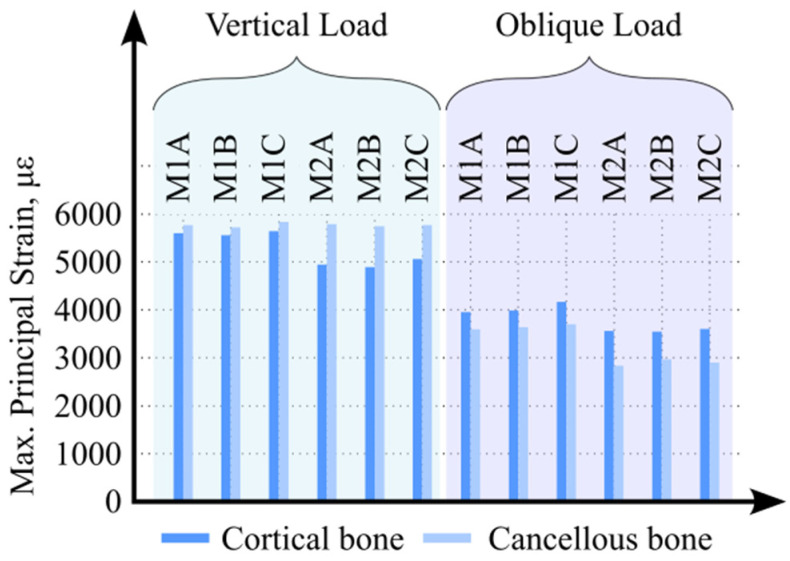
Maximum principal strain values in cortical and cancellous bone under vertical and oblique loading conditions.

The maximum principal strain (tensile strain) values observed in the cortical and cancellous bone under parafunctional vertical and oblique loading conditions are presented in Figure 4. Under vertical loading, comparison of maximum principal strain values revealed that the highest strain occurred in Model M1C, whereas the lowest strain was observed in Model M2B. In all models, the maximum tensile strain values in both cortical and cancellous bone were concentrated on the lingual side of the implant–bone interface.

Under oblique loading conditions, a similar trend was observed. The highest maximum principal strain values in both cortical and cancellous bone were again recorded in Model M1C, while the lowest values were found in Model M2B. In contrast to vertical loading, the maximum tensile strain values under oblique loading were predominantly localized on the buccal side of the implant–bone interface across all models.

To complement the quantitative results presented in Figure 4, color-coded maximum principal strain contour plots are provided to visually demonstrate the strain distribution and concentration areas in the peri-implant bone. These maps facilitate verification of the localization patterns described in the text.

**Figure 5:**
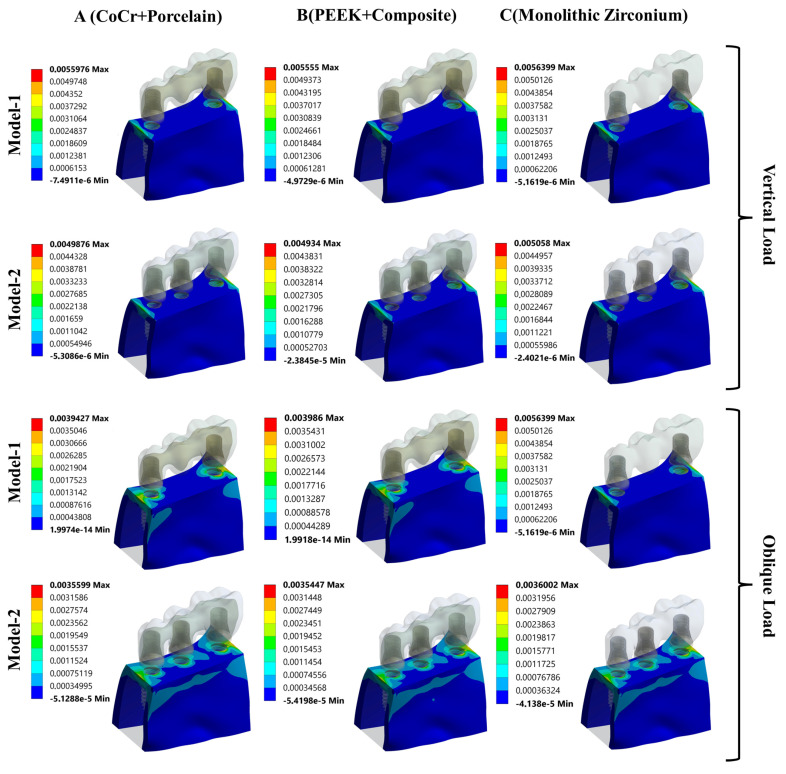
Maximum principal strain distribution in cortical bone under parafunctional vertical and oblique loading conditions.

Figure 5 illustrates the color-coded maximum principal strain distribution in the cortical bone under parafunctional vertical and oblique loading conditions. Under vertical loading, tensile strain concentrations are primarily localized on the lingual side of the implant–bone interface, particularly around the implant neck region. When oblique loading is applied, the strain concentration shifts toward the buccal side across all models, indicating a loading-direction-dependent redistribution of tensile strain in cortical bone.

**Figure 6:**
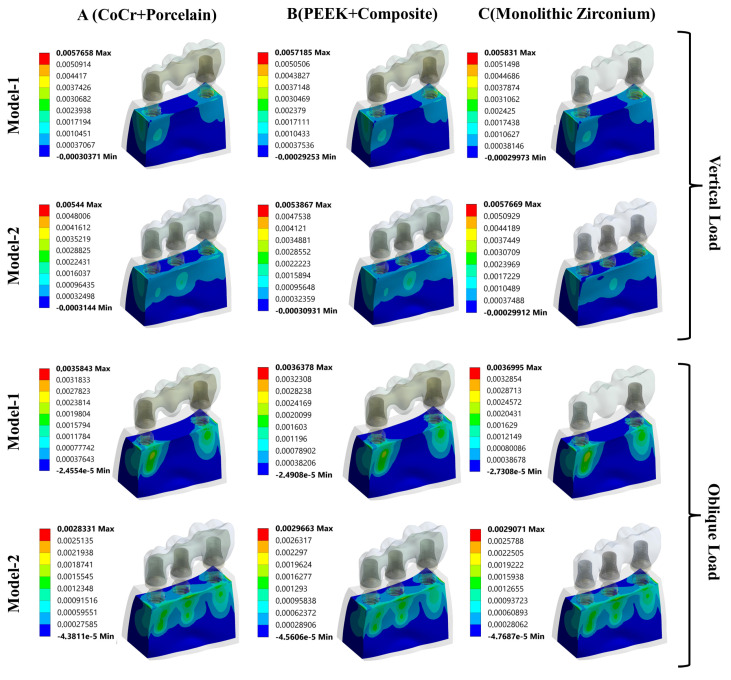
Maximum principal strain distribution in cancellous (trabecular) bone under parafunctional vertical and oblique loading conditions.

Figure 6 presents the maximum principal strain distribution in the cancellous (trabecular) bone under the same loading conditions. Similar to cortical bone, the trabecular bone exhibits higher tensile strain concentrations adjacent to the implant–bone interface. Under vertical loading, strain accumulation is mainly observed on the lingual aspect, whereas oblique loading results in a predominant buccal-side localization.

**Figure 7:**
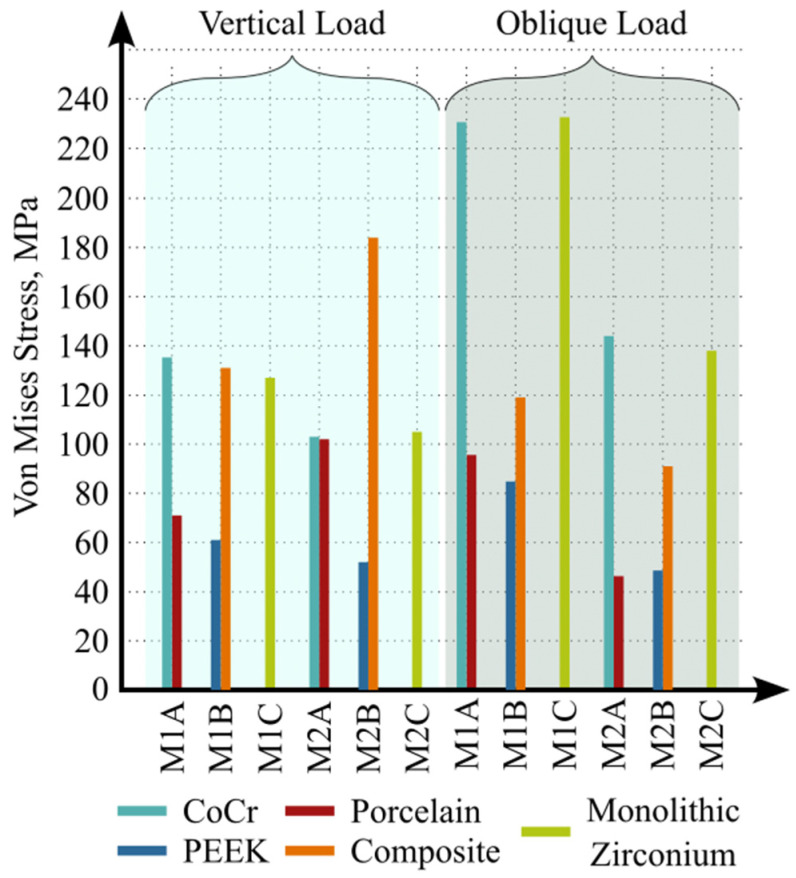
Maximum von Mises stress values in prosthetic superstructure materials under vertical and oblique loading conditions.

The maximum von Mises stress values generated in the prosthetic superstructure materials under parafunctional vertical and oblique loading conditions are shown in Figure 7. Under vertical loading, the highest von Mises stress value was observed in the three-implant-supported PEEK composite veneered model, reaching 184 MPa. The second highest value was recorded in the two-implant-supported monolithic zirconia (MZ) model at 127 MPa. The lowest von Mises stress value was detected in the three-implant-supported PEEK framework, measuring 51 MPa. In the cobalt–chromium (Co–Cr) and monolithic zirconia models, maximum stress concentrations were predominantly located in the connector regions.

Under oblique loading conditions applied to the three-unit bridge models, the highest von Mises stress value was observed in the two-implant-supported monolithic zirconia model, reaching 233 MPa. In the Co–Cr and MZ models, maximum stress concentrations were primarily located in the connector and buccal cervical regions, whereas in the PEEK models, stress concentrations were limited to the buccal cervical regions.

## 4. Discussion

In the present study, stress distributions in implants and prosthetic superstructures, as well as maximum principal strain values in cortical and cancellous bone, were compared across six different finite element models. When different prosthetic superstructure materials were evaluated, the highest von Mises stress values in the implants were observed in Model M1C under oblique loading, whereas the lowest values were recorded in Models M2A and M2B under vertical loading. In addition, regardless of implant number, maximum principal strain values in cortical and cancellous bone under both vertical and oblique loading conditions showed similar distribution patterns across all models.

Based on these findings, the first hypothesis was partially accepted. The use of a low elastic modulus material such as PEEK resulted in reduced stress levels within the prosthetic restoration; however, it did not produce a significant reduction in stress and strain distribution within the implant or surrounding bone. In contrast, increasing the number of implants led to a reduction in stress and strain values in both the implant and peri-implant bone under vertical and oblique loading conditions. Therefore, the second hypothesis was fully accepted.

Bruxism is frequently described as a risk factor for implant failure, as excessive forces transmitted to implant system components during parafunctional activity may lead to biological and biomechanical complications. However, the available literature on this topic remains limited, and most published reports are based on expert opinion or clinical observations rather than controlled biomechanical analyses [30]. Lobbezoo et al. [31] suggested that increasing the number of implants in patients with bruxism may be biomechanically advantageous. Similarly, Chrcanovic et al. [32], in a retrospective study evaluating implant treatment complications in 98 patients with bruxism, reported higher success rates with an increased number of implants. Consistent with these findings, the present study demonstrated that stress and strain values were lower in the three-implant-supported models than in the two-implant-supported models under both vertical and oblique loading conditions.

The selection of restorative materials plays a critical role in determining the long-term clinical success and stability of implants subjected to parafunctional forces such as bruxism. Prosthetic superstructure materials influence the mechanism of stress transmission during function, transferring loads to the prosthetic components, implant, and bone–implant interface [6,7,8]. The structural properties and geometry of implant-supported fixed prostheses significantly affect stress distribution in the implant and surrounding bone. By splinting the implants, prosthetic superstructures facilitate a more balanced distribution of occlusal forces [33].

Previous studies have reported that rigid, non-polymeric materials with high elastic moduli lack shock-absorbing capacity, leading to increased stress accumulation at the bone–implant interface [9]. In contrast, polymeric materials such as PEEK may promote a more uniform transmission of occlusal forces and reduce harmful stress concentrations, which is particularly relevant for patients with bruxism [10]. However, other studies have suggested that the use of rigid materials may be beneficial in reducing stress transmitted to the implant and surrounding bone [34,35].

Lemos et al. [36] evaluated the biomechanical effects of different implant–abutment connections, retention types, and metal–ceramic and monolithic zirconia crowns using finite element analysis and reported no significant differences in stress and strain values in the bone, implant, or implant components based on restorative material. In contrast, Mourya et al. [20] assessed stress distribution under parafunctional loading in Co–Cr–porcelain and PEEK–composite crowns designed for the maxillary first molar region. Their results indicated that Co–Cr frameworks transmitted higher stress to the implant and bone compared with PEEK, while PEEK increased stress in the abutment, potentially leading to screw loosening. Accordingly, the authors recommended PEEK–composite restorations to reduce implant and bone stress in patients with bruxism.

Similarly, Tekin et al. [37] reported that PEEK crowns did not significantly reduce stress in the implant or bone compared with metal-supported ceramic crowns, but did decrease stress in the crown, abutment, and screw. These findings are in agreement with the results of the present study. Sirandoni et al. [38] evaluated the biomechanical behavior of various framework materials, including Co–Cr, titanium, carbon fiber-reinforced PEEK, PEEK, zirconia, and PMMA, and found that PEEK and PMMA reduced stress in implants and abutments while increasing stress in trabecular bone. In contrast, non-polymeric materials such as Co–Cr, titanium, and zirconia demonstrated more favorable stress distributions in peri-implant regions. In the present study, Co–Cr and PEEK exhibited similar stress distributions in implants, abutments, and bone, whereas monolithic zirconia generated stress values approaching the yield strength of titanium in the implant and abutment components.

Nazari et al. [39], in an in vitro study comparing the fracture resistance of Ni–Cr, zirconia, and PEEK frameworks under excessive occlusal loading, reported cohesive–adhesive, cohesive, and adhesive fracture patterns, respectively, and concluded that all three materials could withstand parafunctional forces. Similarly, the present study demonstrates that PEEK provides sufficient resistance against parafunctional loading.

Although PEEK lowered stress concentrations within the prosthetic superstructure, a pronounced reduction in implant or peri-implant bone responses was not observed under the present static, linear-elastic framework, which does not capture viscoelastic energy dissipation commonly implied by dampening in biomimetic narratives [15,40]. From a structural standpoint, the implant–abutment/multiunit joint stiffness and connection geometry may dominate the overall compliance of the system and thereby limit the extent to which a more compliant superstructure material can modify peri-implant stress/strain patterns [41].

The absence of a strong “stress-shielding” effect of PEEK on peri-implant bone in this study can be attributed to system-level stiffness dominance, where the implant–abutment/multiunit joint and metallic components govern overall compliance and constrain how much a more compliant superstructure can reduce bone strain [15,42]. Furthermore, prior FEA investigations on PEEK-containing implant prosthetic systems indicate that peri-implant bone stress/strain reductions are not universal and may remain limited or configuration-dependent, which supports interpreting our findings as comparative estimates under the chosen modeling assumptions [43,44].

von Mises stress values are particularly relevant for ductile materials such as titanium used in implant manufacturing, as they indicate the yield strength beyond which permanent deformation may occur [45]. In this study, the highest von Mises stress value in the implants was observed in the first molar implant of the two-implant-supported monolithic zirconia model, reaching approximately 869 MPa. Notably, the peak von Mises stress (869 MPa) observed in the 2-implant monolithic zirconia model was obtained under intentionally conservative boundary conditions, including extreme static loads of 500 N (oblique) and 1000 N (vertical) selected to approximate an upper-envelope bruxism scenario [46]. In addition, the location of constrained (fixed) bone surfaces is critical in implant FEA because fixing the bone segment at surfaces relatively close to the implants may affect the absolute stress/strain magnitudes, and therefore, these results should be interpreted as worst-case indicators of mechanical demand rather than direct predictions of in vivo yielding [47]. Nevertheless, considering that commonly used implant titanium alloys (e.g., Ti–6Al–4V) present yield-strength ranges that may be approached under high-load conditions, the present finding suggests an elevated overload risk for a 2-implant monolithic zirconia bridge in bruxer-level peak loading and supports the biomechanical advantage of increasing the number of supporting implants. The lowest von Mises stress values were observed in the three-implant-supported Co–Cr and PEEK models, and the critical yield threshold was not exceeded in either the two- or three-implant-supported Co–Cr and PEEK configurations.

Regarding prosthetic materials, von Mises stress values in the Co–Cr framework (yield strength: 460–640 MPa) [48], feldspathic porcelain veneer (yield strength: 150 MPa) [37], PEEK framework (yield strength: 95 MPa) [49], and composite resin veneer (compressive strength: 225 MPa) [50] did not exceed their respective material strength limits. Similarly, in models using monolithic zirconia, stress values in the prosthetic restorations remained below the reported tensile (939 MPa) and compressive (758 MPa) strength limits of zirconia [51].

As in similar studies, potential biomechanical effects in cortical and trabecular bone were evaluated based on maximum principal strain values. According to Frost’s mechanostat approach, strain levels in the bone surrounding the implant neck are generally considered within four ranges: remodeling/repair (200–2500 μɛ), hypertrophy (2500–4000 μɛ), atrophy (≈4000 μɛ), and a fatigue/overload region at higher strain levels (>4000 μɛ) [52]. In this context, the highest principal strain value in our study was obtained in cortical bone under vertical loading, with an average of 5800 μɛ. Although this value is consistent with the fatigue region, it does not, by itself, indicate an inevitable clinical fracture or sudden damage. Rather, it suggests an increased mechanical demand on the bone–implant complex under parafunctional loading.

## 5. Conclusions

Within the limitations of this finite element study, increasing the number of supporting implants under both vertical and oblique loading conditions resulted in reduced stress values in the implants, multiunit abutments, and prosthetic superstructures, as well as decreased strain values in cortical and cancellous bone. The use of different implant-supported prosthetic superstructure materials did not lead to notable differences in bone strain values under either loading condition, regardless of implant number. However, evaluation of the von Mises stress values revealed that the highest stress levels in the implants and multiunit abutments occurred in the first molar implant of the two-implant-supported monolithic zirconia model under oblique loading, reaching levels considered critical in terms of material strength, whereas the PEEK and cobalt–chromium models exhibited similar and comparatively lower stress distributions. Analysis of prosthetic superstructures demonstrated that the lowest stress concentrations were observed in models fabricated with PEEK, which has a lower elastic modulus, while the highest stress values were associated with monolithic zirconia. From a bruxism-related biomechanical perspective, increasing the number of implants and selecting prosthetic superstructure materials with lower elastic moduli may help reduce stress concentrations in both implant components and prosthetic restorations.

## Figures and Tables

**Table 1:** Mechanical properties used in the FEA model.

Material	Elasticity Modulus [MPa]	Poisson’s Ratios	Ref
Titanium (Ti6Al4V)	110,000	0.35	[17]
Cortical bone	13,700	0.3	[17]
Trabecular bone	1370	0.3	[17]
Cobalt–chromium alloy (CoCr)	218,000	0.33	[18]
Feldspathic porcelain	82,800	0.35	[19]
Polyetheretherketone (PEEK)	4000	0.37	[20]
Indirect Resin Composite	50,000	0.3	[21]
Monolithic zirconia	210,000	0.3	[22]
Dual-cure resin cement	6500	0.3	[22]

## Data Availability

The original contributions presented in the study are included in the article, further inquiries can be directed to the corresponding author.
